# The Force-Dependent Mechanism of an Integrin α4β7–MAdCAM-1 Interaction

**DOI:** 10.3390/ijms242216062

**Published:** 2023-11-07

**Authors:** Youmin Su, Zhiqing Luo, Dongshan Sun, Bishan Yang, Quhuan Li

**Affiliations:** 1 School of Bioscience and Bioengineering, South China University of Technology, Guangzhou 510006, China; 202121050567@mail.scut.edu.cn (Y.S.); 202121050573@mail.scut.edu.cn (Z.L.); sundongshan@hec.cn (D.S.);; 2 Guangdong Provincial Engineering and Technology Research Center of Biopharmaceuticals, South China University of Technology, Guangzhou 510006, China

**Keywords:** integrin α4β7, MAdCAM-1, flow-enhanced adhesion, catch bond

## Abstract

The interaction between integrin α4β7 and mucosal vascular addressin cell-adhesion molecule-1 (MAdCAM-1) facilitates the adhesion of circulating lymphocytes to the surface of high endothelial venules in inflammatory bowel diseases (IBDs). Lymphocyte adhesion is a multistep cascade involving the tethering, rolling, stable adhesion, crawling, and migration of cells, with integrin α4β7 being involved in rolling and stable adhesions. Targeting the integrin α4β7–MAdCAM-1 interaction may help decrease inflammation in IBDs. This interaction is regulated by force; however, the underlying mechanism remains unknown. Here, we investigate this mechanism using a parallel plate flow chamber and atomic force microscopy. The results reveal an initial increase in the lifetime of the integrin α4β7–MAdCAM-1 interaction followed by a decrease with an increasing force. This was manifested in a two-state curve regulated via a catch-bond–slip-bond conversion regardless of Ca^2+^ and/or Mg^2+^ availability. In contrast, the mean rolling velocity of cells initially decreased and then increased with the increasing force, indicating the flow-enhanced adhesion. Longer tether lifetimes of single bonds and lower rolling velocities mediated by multiple bonds were observed in the presence of Mg^2+^ rather than Ca^2+^. Similar results were obtained when examining the adhesion to substrates co-coated with chemokine CC motif ligand 25 and MAdCAM-1, as opposed to substrates coated with MAdCAM-1 alone. In conclusion, the integrin α4β7–MAdCAM-1 interaction occurs via ion- and cytokine-dependent flow-enhanced adhesion processes and is regulated via a catch-bond mechanism.

## 1. Introduction

Chronic inflammatory bowel diseases (IBDs), such as Crohn’s disease and ulcerative colitis, are caused by an improper immune response. IBDs are characterized by disrupted lymphocyte adhesion and migration in mesenteric vessels, leading to the uncontrolled recruitment of lymphocytes to the gut [1,2]. The interaction between the adhesion molecule integrin α4β7 in lymphocytes and the mucosal vascular addressin cell-adhesion molecule-1 (MAdCAM-1) in high endothelial venules (HEVs) plays a critical role in this process. Therefore, targeting the interaction between α4β7 and MAdCAM-1 may aid in the development of therapeutic strategies for IBD [3,4,5].

Lymphocyte homing refers to the selective migration of lymphocytes to certain lymphoid organs under normal physiological conditions. This migration is regulated by a multistep process mediated by a cascade of interactions between adhesive molecules, including integrin α4β7, integrin LFA-1, and L-selectin [6]. However, under pathological conditions, lymphocytes are stimulated and uncontrollably recruited to inflammation sites. In such conditions, lymphocytes first attach to and then roll along endothelial cells. Ultimately, the adhesion is stabilized and transmigration to HEVs is achieved [7,8]. This pathological process is mainly mediated by the interaction between integrin α4β7 and MAdCAM-1. Integrin α4β7 is constitutively expressed by naïve T and B cells at relatively low levels [9]. Upon lymphocyte simulation, integrin α4β7 becomes activated and interacts with MAdCAM-1 to mediate lymphocyte rolling and stable adhesion [10,11]. Additionally, integrin α4β7 is also associated with lesions and the progression of atherosclerosis [12], as well as early events following the sexual transmission of the human immunodeficiency virus [13]. MAdCAM-1 is the main ligand of integrin α4β7 and is expressed on endothelia in mucosal tissue and Payer’s patches [14,15], and is aberrantly expressed in extraintestinal endothelial tissues, including those in the joints, eyes, skin, and liver, in IBDs [16].

The adhesion of lymphocytes to HEVs in IBDs depends on the efficient activation of integrin α4β7. Divalent metal ions induce a conformational change in integrin α4β7, thereby increasing its activity [17,18]. The α4 and β7 subunits of the extracellular domain of integrin α4β7 comprise five and eight domains, respectively. The headpieces of these domains are the β propeller of the α4 subunit and the β I domain of the β7 subunit, which contain ligand-binding pockets. The β I domain contains three metal ion-adhesion-dependent regions, namely, the metal ion-dependent adhesion site (MIDAS), adjacent to MIDAS (ADMIDAS), and the ligand-associated metal-binding site (LIMBS). Upon the binding of divalent metal ions, such as Mg^2+^ or Mn^2+^, the ligand-binding pocket of the headpiece of integrin α4β7 opens, enabling effective ligand binding [17]. The binding of integrin α4β7 to MAdCAM-1 facilitates rolling adhesion in the presence of Ca^2+^, Mg^2+^, or Mn^2+^, whereas in other receptor–ligand interactions, such as those between LFA-1 and ICAM-1, α2β1 and collagen, αVβ3 and fibrinogen, and α5β1 and fibronectin, Mg^2+^ and Ca^2+^ exert antagonistic effects, with increasing concentrations of Ca^2+^ inhibiting Mg^2+^-dependent binding [19,20,21,22]. While Ca^2+^ and/or Mg^2+^ ions can increase rolling adhesion via integrin α4β7, the binding of Mg^2+^ or Mn^2+^ ions can activate integrin α4β7 to transition to a higher affinity state, thereby enabling firm adhesion [18]. Extensive mechanistic research on the activation of integrin α4β7 by various metal ions has revealed the roles of chemokines in the activation and ligand-binding process of α4β7 [10,23]. Among these chemokines, chemokine CC motif ligand (CCL) 25 and CCL28 can induce α4β7-dependent lymphocyte arrest on MAdCAM-1 under shear stress [23]. Therefore, these two chemokines may activate integrin α4β7 to a certain extent, leading to ligand binding. CCL25, also known as thymus-expressed chemokine (TECK), acts in conjunction with the chemokine receptor CCR9, which is selectively expressed in the small intestine and colon [24]. Recent studies have identified the interaction of CCL25 and CCR9 in intestinal tissue as an important factor influencing intestinal lymphocyte homing [24,25]. Therefore, it is essential to elucidate the effect of CCL25 on integrin α4β7-mediated lymphocyte adherence.

Lymphocyte adhesion in IBDs not only depends on the integrin α4β7–MAdCAM-1 interaction, but also on the hemodynamics of blood flow [26]. During adhesion, mechanical forces from the extracellular environment can activate cells via integrin-mediated transmembrane transport and regulate the physical interaction between integrins and their ligands. Many adhesive molecules have previously been reported to mediate this flow-enhanced adhesion, a phenomenon where cell adhesion increases with the increasing force [27,28]. This increased adhesion results from the prolonged lifetime of molecular bonds under force. These bonds are referred to as catch bonds [29]. Such interactions have been observed in interactions of selectins with PSGL-1 [30,31], actin with myosin [32], the FimH receptor with mannose [33], hyaluronic acid with CD44 [34], adhesin with peptides [35,36], and the molecular coupling of the glycocoib protein Ibα with the von Willebrand factor [37]. Although catch-bond interactions also exist in the integrin family [22], whether integrin α4β7 exhibits the same flow-enhanced rolling adhesion remains unclear, necessitating a further exploration into the existence of a catch-bond mechanism to regulate this adhesion phenomenon.

Here, we investigate the mechanism of cell adhesion mediated by the integrin α4β7–MAdCAM-1 interaction using a parallel plate flow chamber (PPFC) and an atomic force microscope (AFM). The dynamics of the integrin α4β7–MAdCAM-1 interaction are analyzed under various force conditions and distinct ionic conditions. The transient lifetime and rolling parameters of integrin α4β7 are measured using the PPFC. The lifetimes of individual integrin α4β7–MAdCAM-1 bonds under different forces are measured via AFM experiments. The findings of this study elucidate the force-regulated mechanism of cell adhesion mediated by the specific binding of integrin α4β7 and MAdCAM-1 and may aid in the subsequent discovery or design of drugs for the treatment of IBDs.

## 2. Results

### 2.1. Specificity of the Integrin α4β7–MAdCAM-1 Interaction

**Figure 1:**
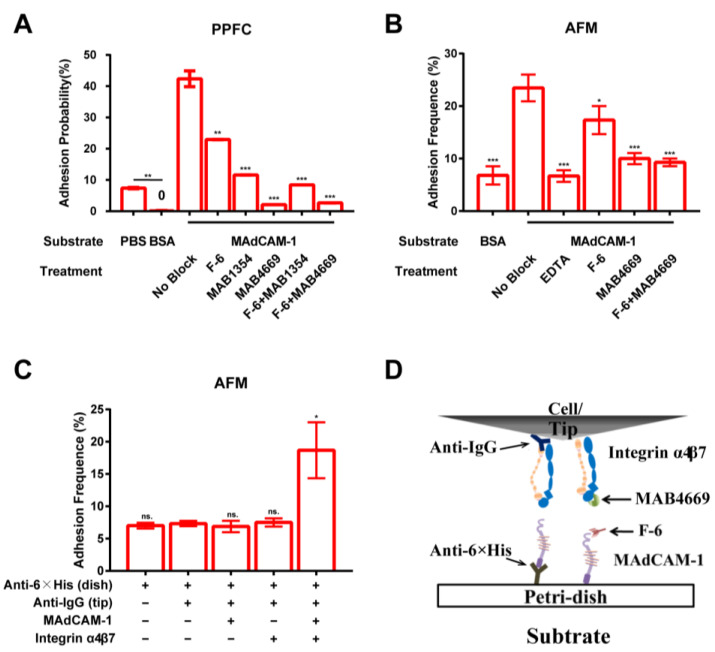
Binding specificity of the interaction between integrin α4β7 and mucosal vascular addressin cell-adhesion molecule-1 (MAdCAM-1). (**A**) Adhesion probability of integrin α4β7-expressing RPMI 8226 cells (5 × 10^5^ cells/mL, suspended in Hank’s balanced salt solution (HBSS) with 2% bovine serum albumin (BSA) and 1 mM Ca^2+^) adhered to MAdCAM-1-coated (2 μg/mL) substrates in a parallel plate flow chamber (PPFC) at a shear stress of 0.3 dyn/cm^2^ for 1 min. The 35 mm dishes coated with PBS or HBSS alone with 2% BSA on the substrate of the flow chamber were used as negative controls. To test adhesion, the dishes were functionalized with MAdCAM-1 (2 μg/mL). The dishes were incubated with F-6 (anti-MAdCAM-1 monoclonal antibody, mAb), MAB1354 (anti-α4 mAb), and MAB4669 (anti-β7 mAb), and used as blocking controls. The PBS group was compared with the BSA group, and all experimental groups coated with MAdCAM-1 were compared with the no-block group. (**B**) Adhesion frequency of integrin α4β7 physically adsorbed on an atomic force microscope (AFM) tip and MAdCAM-1 adsorbed on a Petri dish in buffer containing a 2% BSA with or without ethylenediaminetetraacetic acid (EDTA). The blocking treatments were the same as in the flow chamber experiments. All experimental groups were compared with the no-block group. (**C**) Molecular orientation detection test. Anti-IgG and anti-6xHis antibodies were incubated on an AFM tip and Petri dish, respectively, to capture integrin α4β7 and MAdCAM-1, and the adhesion frequency was measured. (**D**) Schematic of PPFC and AFM substrate functionalization. All data are presented as the mean ± SD from three independent experiments, except for the antibody blocking treatment in the PPFC (due to antibody depletion). The *p*-values from unpaired two-tailed Student’s *t*-tests were used to indicate the statistical significance of the data (ns. for no significant difference, * for *p* < 0.05, ** for *p* < 0.01, *** for *p* < 0.001).

To investigate the specificity of the integrin α4β7–MAdCAM-1 interaction, RPMI 8226 cells expressing integrin α4β7 were pumped into the PPFC and allowed to adhere to the MAdCAM-1-coated substrate. The adhesion probability was calculated by dividing the number of observed adhesion events within 3 min by the total number of cells flowing through the field of view. Only a small number of cells was found to adhere to the phosphate-buffered saline (PBS)-covered substrate due to non-specific interactions (Figure 1A, Column 1). Nevertheless, this interaction was abrogated upon blocking the substrate with PBS containing 2% bovine serum albumin (BSA) incubated at 4 °C for 2 h, and resulted in a reduction in the adhesion probability from 8% to almost 0% (Figure 1A, Column 2). In the adhesion experiment, the adhesion probability increased by up to 40% for integrin α4β7-expressing RPMI 8226 cells that adhered to the MAdCAM-1-coated substrate (Figure 1A, Column 3). To investigate whether cell adhesion was specifically mediated by the integrin α4β7–MAdCAM-1 interaction, the cells were incubated with anti-integrin α4 (MAB1354) and anti-integrin β7 (MAB4669) antibodies, and the anti-MAdCAM-1 antibody (F-6) was also added to the substrate. Consequently, the cell-adhesion probability was reduced to approximately 20% (Figure 1A, Column 4–6). Furthermore, the adhesion of RPMI 8226 cells to the MAdCAM-1-coated substrate was significantly reduced by adding anti-integrin β7 and anti-integrin α4 antibodies, with or without F-6 (Figure 1A, Column 7,8). These results indicate that the adhesion of RPMI 8226 cells via the integrin α4β7–MAdCAM-1 interaction is specific. The presence of multiple binding sites on MAdCAM-1 potentially contributed to the interaction with integrin α4β7, facilitating the adhesion of RPMI 8226 cells (Figure 1A, Column 4). However, all adhesion events were exclusive to integrin α4β7, regardless of the uncertainty regarding the binding sites on MAdCAM-1.

An AFM analysis was also performed to investigate the specificity of the integrin α4β7–MAdCAM-1 interaction. In the absence of integrin α4β7, the adhesion frequency of non-functionalized AFM tips blocked only with BSA was found to be lower than 8% (Figure 1B, Column 1). In contrast, the adhesion frequency was found to increase by up to 23% when the AFM tips were coated with both integrin α4β7 and BSA (Figure 1B, Column 2). However, the adhesion frequency decreased significantly when the F-6 antibody and/or integrin β7 antibody (MAB4669) or the metal ion-chelating agent ethylenediaminetetraacetic acid (EDTA) was added to the solution (Figure 1B, Column 3–6)). This indicated that the interaction between integrin α4β7 and MAdCAM-1 was specific and dependent on metal ions.

Furthermore, we used an antibody capture method to assess the adhesion frequency of integrin α4β7 and MAdCAM-1 coated on the tips and dishes (Figure 1C,D) to investigate the effect of molecular orientation on adhesion. The results showed that the adhesion frequency of integrin α4β7 and MAdCAM-1 was approximately 20% when the target molecule was captured by an antibody with an aligned molecular orientation (Figure 1C, Column 5). This was similar to the adhesion frequency of physical adsorption with diversified molecular orientation (Figure 1B, Column 2). This demonstrated that physical adsorption did not influence the interaction between integrin α4β7 and MAdCAM-1. Therefore, the direct physical adsorption of integrin α4β7 and MAdCAM-1 was used to functionalize the AFM and PPFC analyses in subsequent experiments.

In the PPFC (Figure 1A) and AFM (Figure 1B) experiments, cell adhesion could not be completely blocked with the F-6 antibody, possibly due to the non-unique binding sites of MAdCAM-1 to integrins. A previous study reported that the key integrin binding site in MAdCAM-1 was located in the proximal N-terminal IgSF domain (D1), in which Asp42, a key residue for integrin binding, was located in the CD loop [38,39]. Asp42 is a vital residue in the recognition region of the F-6 antibody and is located between five and 43 near the N-terminus of MAdCAM-1. Furthermore, another binding site for integrin exists in the N-terminal IgSF domain of MAdCAM-1. The IgSF domain (D2) is the second domain involved in integrin binding [38,39], and the D2 domain of MAdCAM-1 contains a negatively charged β band that extends far from the domain [40]. This β band can directly participate in integrin binding and optimize the structure of D1 and D2, which is conducive to the adhesion of MAdCAM-1 to integrin [41]. Therefore, the two alternative binding sites of MAdCAM-1 for integrins could explain the incomplete blocking of MAdCAM-1 with antibody F-6.

### 2.2. Binding Affinity of Integrin α4β7 with MAdCAM-1 Remains Consistent in the Presence of Various Metal Ions

**Figure 2:**
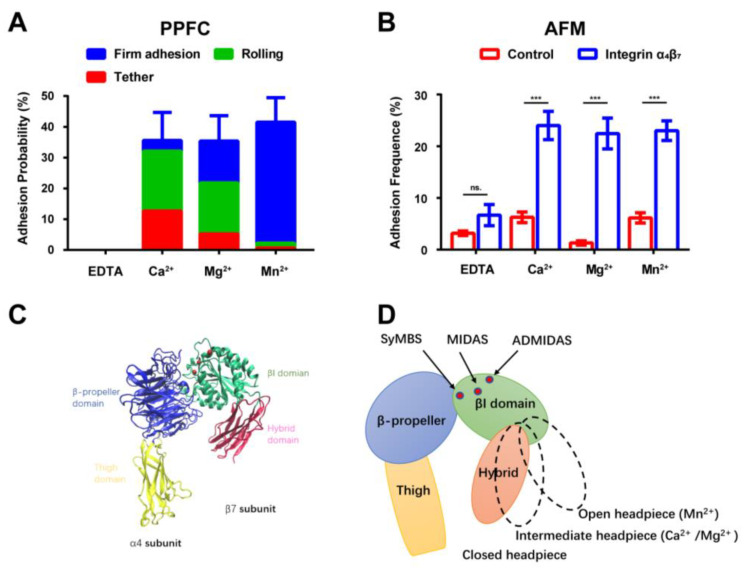
Adhesion of integrin α4β7 to mucosal vascular addressin cell-adhesion molecule-1 (MAdCAM-1) in the presence of different ion types. (**A**) Adhesion probability of RPMI 8226 cells tethering, rolling, and firmly adhering to MAdCAM-1-coated (2 μg/mL) parallel plate flow chamber (PPFC) substrates were measured at a shear stress of 0.3 dyn/cm^2^. Adhesion events in the presence of different ion types were calculated by adding different metal cations (1 mM Ca^2+^, 1 mM Mg^2+^, 0.5 mM Mn^2+^, and 10 mM ethylenediaminetetraacetic acid (EDTA)) to cell suspensions. The data were collected from the cell-adhesion events within the first 3 min in the same field of view. Data are shown as mean ± SD (*n* = 3). (**B**) Adhesion frequency of integrin α4β7 interacting with MAdCAM-1 in the presence of various metal ions (Ca^2+^, Mg^2+^, Mn^2+^). No integrin α4β7 was used in the control. Data are shown as mean ± SD (*n* = 3). The *p*-values from unpaired two-tailed Student’s *t*-tests were used to indicate the statistical significance of the data (ns. for no significant difference, *** for *p* < 0.001). (**C**) Overall structure of the α4β7 headpiece in the closed, low-affinity state (structure from PDB: 3V4P [14], visualized using VMD). (**D**) Schematic depiction of integrin-headpiece domain organization and conformational states. The closed, intermediate, and open headpieces bind with Ca^2+^ and/or Mg^2+^ and Mn^2+^.

Most integrin proteins depend on metal ions to interact with their respective ligands, such as αLβ2, αvβ3, and α5β1. To investigate the metal ion dependency of the adhesion of α4β7-expressing RPMI 8226 cells to MAdCAM-1, we performed adhesion experiments using different ion solutions, including 1 mM Ca^2+^, 1 mM Mg^2+^, and 0.5 mM Mn^2+^. The PPFC results revealed that the integrin α4β7-expressing RPMI 8226 cells did not adhere to the MAdCAM-1-coated substrate when EDTA was added to the cell solution to chelate the metal ions (Figure 2A). Moreover, the adhesion probability was low, indicating ion dependency. However, the addition of Ca^2+^, Mg^2+^, and Mn^2+^ ions increased the adhesion probability to approximately 35% (Figure 2A). In the presence of Ca^2+^ or Mg^2+^ ions, most adhering cells tethered and rolled on the bottom of the PPFC; however, in the presence of Mn^2+^, cells firmly adhered to the MAdCAM-1-coated substrate. These results indicate that the MAdCAM-1–integrin α4β7 bonds mediating the adhesion behavior of cells are ion-dependent. In the presence of Ca^2+^ or Mg^2+^, integrin α4β7 mainly mediated the tethering and rolling adhesion of RPMI 8226 cells, whereas in the presence of Mn^2+^, it mainly mediated the stable adhesion of cells, consistent with the findings of previous studies [18]. An AFM experiment was performed to confirm this finding. The adhesion frequency of integrin α4β7 to MadCAM-1 was measured under various metal ionic environments by adding Ca^2+^, Mg^2+^, and Mn^2+^ to the solution (Figure 2B). The results showed an ion-dependent interaction, as the adhesion frequency decreased to non-specific adhesion levels. No significant differences between the adhesion frequencies of integrin α4β7–MAdCAM-1 interactions in different ion solutions were observed, with all treatments resulting in up to a 20% adhesion, consistent with the PPFC measurements. Moreover, considering the co-existence of Ca^2+^ and Mg^2+^ under physiological conditions, we performed adhesion probability and rolling adhesion experiments using Ca^2+^ and Mg^2+^ simultaneously (Figure S1). The adhesion probability results showed that most of the RPMI 8226 cells tethered to or rolled on the MAdCAM-1 substrate, similar to the adhesion observed under the Ca^2+^-only stimulation, indicating that integrin α4β7 was in the low-activity state.

To elucidate the influence of different ions on the integrin α4β7 conformation, we downloaded crystal structure data for integrin α4β7 in complex with metal ions from the PDB (Figure 2C). Previous studies reported that both the rolling adhesion mediated by a closed-headpiece, low-affinity state of α4β7 and firm adhesion mediated by an open-headpiece, high-affinity state of α4β7 required an intact MIDAS in the βI domain, which was hypothesized to hold an Mg^2+^ ion [14]. The synergistic metal ion-binding site (SyMBS) and ADMIDAS showed different affinities for different metal ions (Ca^2+^, Ca^2+^/Mg^2+^, Mg^2+^, or Mn^2+^). Accordingly, high-, intermediate-, and low-affinity states corresponded to open, intermediate, and closed conformations, respectively (Figure 2D). Studies of metal ions binding to integrin α4β7 have reported complex functions. Integrin α4β7 contains three metal ion-binding sites, namely, LIMBS, MIDAS, and ADMIDAS (Table S1). Both Mg^2+^- and Mn^2+^-dependent firm adhesions and Ca^2+^-dependent rolling adhesion require metal ion binding at MIDAS to interact with MAdCAM-1 [17]. However, ADMIDAS is a negative regulatory site that mainly mediates rolling adhesion. Notably, a high concentration of Ca^2+^ exerts a negative regulatory effect at this site by blocking the I-like domain, causing Mn^2+^ to compete with Ca^2+^ for ADMIDAS and thus activating integrins [17,42]. Conversely, SyMBS acts as a positive regulatory site that favors stable adhesion. Moreover, the synergy between Ca^2+^ and Mg^2+^ at low concentrations arises from their binding to SyMBS and MIDAS, respectively [17].

### 2.3. Transition of Catch–Slip Bonds Govern the Lifetime of Interactions below and above the Optimal Force

**Figure 3:**
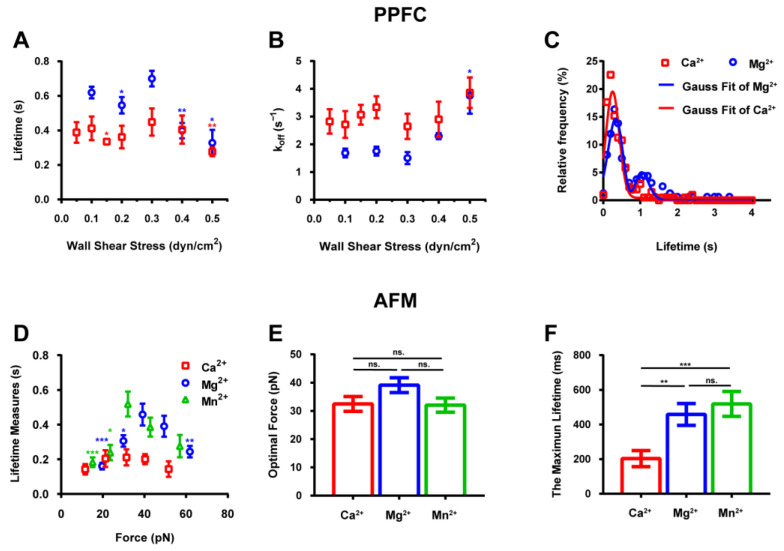
Measurement of interaction lifetime using a parallel plate flow chamber (PPFC) and an atomic force microscope (AFM). (**A**) The tether lifetime curves align with wall shear stress. Tether lifetimes in the presence of Ca^2+^ and Mg^2+^ plotted against shear stress for the RPMI 8226 cells that transiently tether to low-density mucosal vascular addressin cell-adhesion molecule-1 (MAdCAM-1) (substrate coated with the 0.25 μg/mL MAdCAM-1 solution). (**B**) Dissociation rate (*k_off_*) in the presence of Ca^2+^ or Mg^2+^ ions at different shear stresses. For each metal ion, all experimental groups were compared with the group with a wall shear stress of 0.3 dyn/cm^2^. (**C**) Distribution of the number of adhesion events under the representative shear stress of 0.3 dyn/cm^2^. (**D**) Lifetime–force curves of the integrin α4β7–MAdCAM-1 interaction in Ca^2+^, Mg^2+^, and Mn^2+^ ionic solutions. (**E**) Optimal force and (**F**) maximum lifetime of the integrin α4β7–MAdCAM-1 interaction. The data in (**A**–**D**) and (**D**–**F**) were obtained from three independent PPFC and AFM experiments. The *p*-values from unpaired two-tailed Student’s *t*-tests were used to indicate the statistical significance of the data (ns. for no significant difference, * for *p* < 0.05, ** for *p* < 0.01, *** for *p* < 0.001).

Previous studies have indicated that the interactions of integrins and ligands are regulated by force [43]. Here, we investigated whether the association and dissociation of integrin α4β7 and MAdCAM-1 were also regulated by force. To investigate this, we measured the force dependence of the average lifetime of individual integrin α4β7–MAdCAM-1 interactions in different ionic solutions using a PPFC and AFM (Figure 3). Using the PPFC, we measured the transient tether events of RPMI 8226 cells treated with 1 mM Ca^2+^ or 1 mM Mg^2+^ on a substrate coated with low-density MAdCAM-1 (0.25 μg/mL) under flow. The cells did not roll or skip on the flow chamber bottom functionalized with low-density MAdCAM-1. By obtaining the single molecular lifetime of each tether event, the mean tether lifetime of RPMI 8226 cells was measured and found to display a biphasic pattern in the presence of Ca^2+^ ions (Figure 3A). Figure 3A shows that the tether lifetime of cells first increases and then decreases with an increase in the wall shear stress, indicating a catch–slip-bond interaction mechanism. This biphasic mode of the tether lifetime showed a phase-transitional process from catch to slip bonds, as first described by Finger et al. [44]. The optimal shear stress threshold was found to be 0.3 dyn/cm^2^. A similar biphasic mode was observed for Mg^2+^, although the tether lifetime curve shifted slightly upward, suggesting that Mg^2+^ ions activate integrin α4β7 more strongly than Ca^2+^ ions (Figure 3A). The inverse relationship between the transient tether lifetime curve and the dissociation rate (*k_off_*) curve indicated that the catch–slip bond mechanism regulates the interaction of integrin α4β7 with its ligand, MAdCAM-1 (Figure 3B). To better understand the long lifetime and low dissociation rates of Mg^2+^ ions, we further analyzed the frequency distribution of the tether lifetime for the representative shear stress of 0.3 dyn/cm^2^ (Figure 3C). The frequency distribution histograms for the presence of Ca^2+^ or Mg^2+^ ions were nearly the same (bimodal), and the Gauss fitting followed the normal distribution. However, the second peak of the tether lifetime was higher in the presence of Mg^2+^ than it was for Ca^2+^. Therefore, we hypothesized that integrin α4β7 expressed by the RPMI 8226 cells was activated in the presence of Mg^2+^ ions, resulting in a prolonged tether adhesion to MAdCAM-1.

The AFM results show that the lifetime of the integrin α4β7–MAdCAM-1 interaction increases initially and then decreases with an increasing force, showing a two-phase transition for Ca^2+^, Mg^2+^, and Mn^2+^ (Figure 3D). The curve shifts towards the left and downwards for Ca^2+^ compared to the curve position observed for the other ions. Subsequently, we determined the optimal force (Figure 3E) and the maximum lifetimes (Figure 3F) in the three ionic environments. We found no significant differences in the optimal force, whereas the maximum lifetime was the shortest in the presence of Ca^2+^. Our findings indicate that the same catch-bond mechanism regulates the integrin α4β7–MAdCAM-1 interaction in the presence of different ions. However, the lifetime of the bond was found to be influenced by differences in the structural affinities of integrins under different ionic conditions.

### 2.4. Rolling Behaviors of RPMI 8226 Cells Mediated by the Integrin α4β7–MAdCAM-1 Interaction under Flow

**Figure 4:**
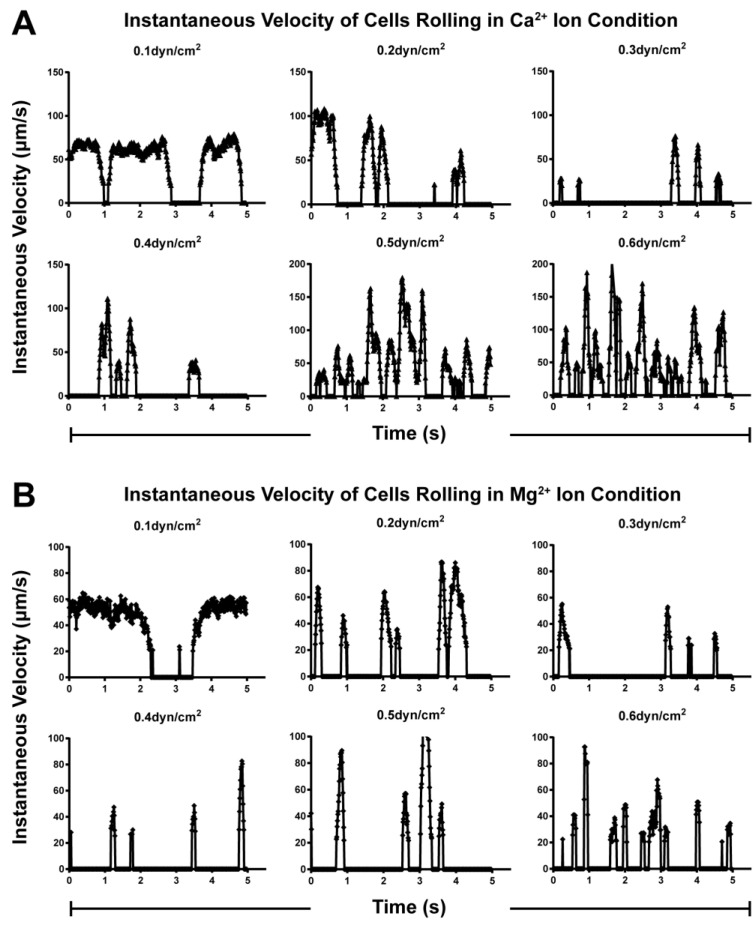
Instantaneous rolling velocities of cells in the presence of Ca^2+^ and Mg^2+^ ions. Six representative instantaneous velocities of RPMI 8226 cells rolling on high-density mucosal vascular addressin cell-adhesion molecule-1 (MAdCAM-1) (2 μg/mL) at each shear stress level in the presence of Ca^2+^ (**A**) and Mg^2+^ (**B**) ions. The wall shear stresses were 0.1, 0.2, 0.3, 0.4, 0.5, and 0.6 dyn/cm^2^. The instantaneous velocities were set as zero when their values were below 25 μm/s.

The abovementioned two-phase transition of lifetime was regulated by mechanical force, where the integrin α4β7-expressing RPMI 8226 cells tethered to the low-density MAdCAM-1 via a single-bond interaction. However, it was unclear whether the rolling process mediated by multi-bond interactions was also regulated by the flow force. To clarify this, we analyzed the rolling behaviors of RPMI 8226 cells on a high-density MAdCAM-1 substrate. The rolling process of RPMI 8226 cells was segregated into stop and go phases due to the formation and breakage of intermolecular bonds, consistent with a previously established model [43]. Here, a rolling step is referred to as a cycle of acceleration and deceleration above a threshold that can be distinguished from the noise caused by fluctuations in RPMI 8226 cells. Six typical instantaneous velocity profiles of RPMI 8226 cells rolling on MAdCAM-1-coated substrates were observed for 5 s at various wall shear stresses of 0.1, 0.2, 0.3, 0.4, 0.5, and 0.6 dyn/cm^2^ in the presence of Ca^2+^ (Figure 4A) and Mg^2+^ (Figure 4B) ions. These instantaneous velocity profiles indicated a possible force-dependent cell-rolling behavior. An increase in shear stress promoted the transition between the stop and go phases, where the rolling step changed from occasional to continuous periodic events in the presence of Ca^2+^ or Mg^2+^ ions (Figure 4A,B). The rolling velocity decreased to 0.1 and 0.3 dyn/cm^2^ and then increased to 0.3 and 0.6 dyn/cm^2^. In contrast, the stop durations of rolling cells increased first and then decreased as the shear stress increased from 0.1 to 0.6 dyn/cm^2^. At a low shear stress of 0.1 dyn/cm^2^, RPMI 8226 cells rarely stopped at the bottom, and they slid along rather than rolled on the substrate. When the shear stress reached 0.3 dyn/cm^2^, the rolling of RPMI 8226 cells was the most stable. Therefore, the most ordered rolling behavior was observed in the presence of Ca^2+^ or Mg^2+^ ions. These conditions were determined to be optimal shear stress conditions, and rolling became disordered beyond this level. The same rolling behavior was observed in Ca^2+^ and Mg^2+^ ion solutions. The cells rolled more slowly in the solution containing Mg^2+^ than in the solution containing Ca^2+^, possibly because the integrin α4β7 on the cell was activated by Mg^2+^ ions to the intermediate affinity state, thus binding strongly to MAdCAM-1 and mediating the slow rolling of cells on the substrate.

### 2.5. Ion and Force Dependence of RPMI 8226 Cell-Rolling Adhesion on MAdCAM-1-Coated Substrate under Flow

**Figure 5:**
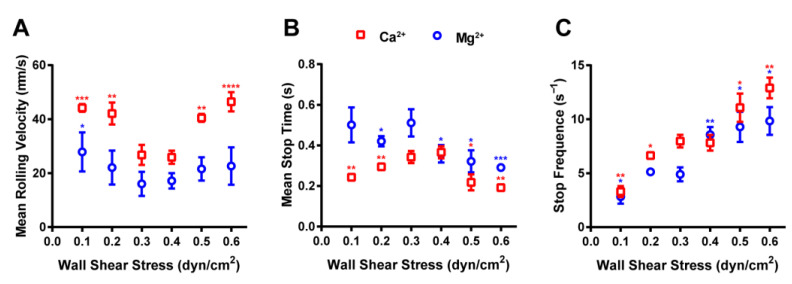
Quantitative analysis of the rolling process. (**A**) Rolling velocity, (**B**) mean stop time, and (**C**) stop frequency of RPMI 8226 cell rolling plotted against wall shear stress. RPMI 8226 cells were perfused into the flow chamber to interact with mucosal vascular addressin cell-adhesion molecule-1 (MAdCAM-1) (2 μg/mL) under shear stress levels ranging from 0.1 to 0.6 dyn/cm^2^. Cells were suspended in 1 mM Ca^2+^ (red boxes) or 1 mM Mg^2+^ (blue circles) ions. The data were recorded at 100 fps and represent the mean ± SD of three replicates. For each metal ion, all experimental groups were compared with the group with a wall shear stress of 0.3 dyn/cm^2^. *p*-values obtained from unpaired two-tailed Student’s *t*-tests were used to indicate the statistical significance of the data (* for *p* < 0.05, ** for *p* < 0.01, *** for *p* < 0.001, **** for *p* < 0.0001).

**Figure 6:**
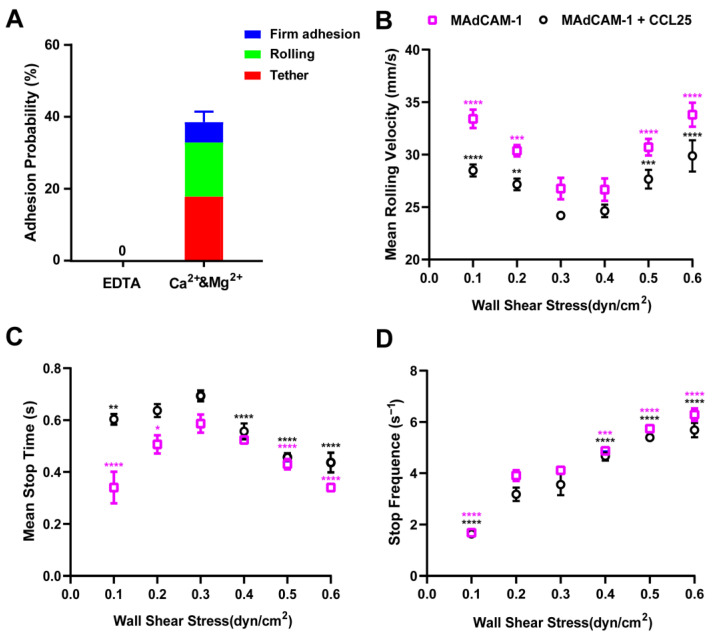
Adhesion probability and quantitative analysis of the rolling process of RPMI 8226 cells on chemokine CC motif ligand (CCL) 25 and/or mucosal vascular addressin cell-adhesion molecule-1 (MAdCAM-1) in the presence of Ca^2+^ and Mg^2+^. RPMI 8226 cells were suspended in 1 mM Ca^2+^ and 1 mM Mg^2+^. (**A**) Adhesion probabilities of RPMI 8226 cells tethering, rolling, and firmly adhering onto MAdCAM-1 substrates were measured at a shear stress of 0.3 dyn/cm^2^, with an additional group co-stimulated by 1 mM Ca^2+^ and 1 mM Mg^2+^. Data are shown as mean ± SD (*n* = 3). (**B**) Mean rolling velocity, (**C**) mean stop time, and (**D**) stop frequency of rolling of RPMI 8226 cells on different substrates plotted against wall shear stress levels under 1 mM Ca^2+^ and 1 mM Mg^2+^ conditions. Cells were perfused into the flow chamber at shear stresses ranging from 0.1 to 0.6 dyn/cm^2^, interacting with MAdCAM-1 (4 μg/mL) and CCL25 (1 μg/mL) (black circles) or MAdCAM-1 (4 μg/mL) alone (pink boxes). The data were recorded at 100 fps and represent the mean ± SD of three experiments. For the analysis of the rolling process, all experimental groups were compared with the group with a wall shear stress of 0.3 dyn/cm^2^. The *p*-values of the unpaired two-way analysis of variance for multiple comparisons were used to indicate the statistical difference significance of the data (* *p* < 0.05, ** *p* < 0.01, *** *p* < 0.001, **** *p* < 0.0001).

To quantitatively assess the abovementioned observations, we measured the mean velocities of rolling cells under various wall shear stress levels. As shown in Figure 5A, with an increasing wall shear stress, the mean velocity of rolling cells in the presence of Ca^2+^ and Mg^2+^ ions decreases first and then increases at the shear threshold of approximately 0.3 dyn/cm^2^. Moreover, the mean rolling velocity of cells treated with Mg^2+^ alone was slower than that of cells treated with Ca^2+^ alone. This result indicates that the behavior of cells rolling on MAdCAM-1 exhibits a flow-enhanced rolling phenomenon and a two-phase rolling pattern, as the wall shear stress increases (Figure 5A). In the presence of Ca^2+^, the cells rolled faster, indicating that the affinity of integrin α4β7 in the presence of Mg^2+^ was higher than that in the presence of Ca^2+^. Therefore, integrin α4β7 may be activated by Mg^2+^ ions to a higher affinity state. Furthermore, considering that Ca^2+^ and Mg^2+^ have been used in some studies to co-stimulate integrin, we also conducted cell-rolling experiments with both Ca^2+^ and Mg^2+^ (Figure 6B). The flow-enhanced rolling phenomenon and the two-phase rolling pattern were also observed under the coordinated stimulation of Ca^2+^ and Mg^2+^. Moreover, we found that the level of integrin activation by Ca^2+^ and Mg^2+^ together fell between the levels observed for Ca^2+^ and Mg^2+^ alone. In particular, the rolling velocity under co-stimulation with Ca^2+^ and Mg^2+^ showed a biphasic curve and almost overlapped with the curve observed under stimulation with Ca^2+^ alone.

We also measured the mean stop time and stop frequency for the rolling mediated by the interaction of integrin α4β7 and MAdCAM-1 under force. Here, the mean stop time is defined as the average duration of each stop period of a cell during the rolling process, and stop frequency is defined as the number of RPMI 8226 cells stopped in the rolling process within 5 s. We plotted the values for the two rolling parameters against the wall shear stress scaled to the same range as the rolling velocity and tether lifetime to examine the rolling stabilization of flowing RPMI 8226 cells under two conditions (the presence of Ca^2+^ or Mg^2+^ ions). The results show that the mean stop time first increases and then decreases as the shear stress increases (Figure 5B). The curve of the mean stop time was complementary to the mean rolling velocity curve (Figure 5A), but in line with the tether lifetime curve (Figure 3A). The stop frequency increased monotonically with the increased shear stress (Figure 5C), which may have occurred due to the shortening of the process of integrin α4β7–MAdCAM-1 anterior bond dissociation and posterior bond formation as the shear stress increased in the presence of Ca^2+^ and Mg^2+^. These results indicate the presence of a mechanism driven by the increase in single-bond tether lifetimes resulting from an increased shear stress level, which in turn increased the rolling stop time of the cells and decreased the rolling velocity. At the optimal shear stress threshold (0.3 dyn/cm^2^), the mean stop time in the presence of Mg^2+^ was slightly higher than that in the presence of Ca^2+^. A similar trend was also observed for the tether lifetime.

These results suggest that cell rolling is governed by the formation and dissociation of bonds between integrin α4β7 and MAdCAM-1. The integrin α4β7-mediated flow-enhanced rolling adhesion of RPMI 8226 cells was found to be regulated by a catch-bond mechanism. Under shear stress, the catch bond increases the strength of the interaction between integrin α4β7 and MAdCAM-1 and the lifetime of the integrin α4β7–MAdCAM-1 single bond under shear stress conditions. This, in turn, increases the stop time of rolling cells, decreases the rolling velocity, and improves the order of cell rolling.

### 2.6. Rolling Adhesion of RPMI 8226 Cells on the MAdCAM-1- and/or CCL25-Coated Substrate

The effects of various metal ions on integrin activation were extensively examined. In addition to metal ions, chemokines also play an important role in integrin activation. In this study, Ca^2+^ and Mg^2+^ metal ions were studied separately, and both appeared to co-stimulate integrin α4β7 (Figure 6A). Furthermore, a new stimulating factor, the chemokine CCL25, was added to simulate physiological and pathological conditions. In this new set of experimental groups, CCL25 and MAdCAM-1 were coated individually or together on the substrate.

Using the same method, we measured the mean rolling velocity, mean stop time, and stop frequency of the rolling cells at gradually increasing wall shear stress levels. With an increase in the shear stress, all three parameters of the rolling cells generally followed consistent trends, regardless of whether the substrate was coated with CCL25. Stimulation with CCL25 did not alter the flow-enhanced rolling phenomenon or the two-phase rolling pattern of lymphocytes; however, the overall rolling velocity of RPMI 8226 cells was reduced after the CCL25 stimulation (Figure 6B). Moreover, the stop time of cell rolling increased (Figure 6C), particularly at low shear stress levels.

Similar changes were observed between the Mg^2+^ activation of α4β7 and Ca^2+^ activation, suggesting that CCL25 could further activate integrin α4β7 under the existing metal ion stimulation, thereby causing changes in the rolling adhesion behavior of RPMI 8226 cells (Figure 6B–D). Sun et al. [10] reported that the proportion of lymphocytes with firm adhesion significantly increased after stimulation with CCL25. Our findings suggest that CCL25 may reduce cell-rolling behavior by further enhancing lymphocyte integrin activity, thereby considerably increasing cell adhesion. Therefore, we propose that the partial mechanisms where the chemokine CCL25 and metal ions activate α4β7 and interfere with cell behavior are similar. This similarity may also extend to other chemokines and integrins.

## 3. Discussion

Here, we used PPFC and AFM techniques to investigate the underlying mechanism of the interaction between integrin α4β7 and MAdCAM-1. In the PPFC experiments, RPMI 8226 cells were perfused to a MAdCAM-1-coated substrate to simulate the capture of flowing cells by MAdCAM-1 on HEVs. Similarly, the interaction of integrin α4β7 with MAdCAM-1 was measured via an AFM analysis under different force conditions. The results show that the lifetimes of single bonds are similar between the PPFC and AFM experiments. The lifetime of the MAdCAM-1–integrin α4β7 interaction first increased and then decreased with an increasing force, indicating a transition from a catch-bond to slip-bond mechanism. Previous studies showed that many integrins and their ligands, such as α5β1, αLβ2, α4β1, and αMβ2, exhibit this two-phase transition behavior [22,45,46,47]. The present study is the first to report the flow-enhanced adhesion and catch-bond regulation of the interaction between integrin α4β7 and MAdCAM-1.

We also investigated the rolling behavior of cells mediated by multiple bonds. The rolling behavior under flow is controlled by the balance between the formation of the front bond and the dissociation of the trail bond, both of which are regulated by biophysical principles. To explore the mechanism involved in this process, we used a previously developed model of rolling, assuming that rolling was mediated via bond alternation and the condition that the front bond must form before the rear bond dissociates. This model was supported by a comparison between the transient tether lifetime (Figure 3A) and the rolling stop time (Figure 5B). We found that both single- and multi-bond models showed a similar trend, characterized by the transition from a catch bond to a slip bond, and the rolling parameters of the model showed that the interaction between integrin α4β7 and MAdCAM-1 was force-dependent. The quantitative analysis results show that the mean stop time (Figure 5B) is consistent with the tether lifetime (Figure 3A), but complementary to the mean rolling velocity curve (Figure 5A). This suggests that integrin α4β7 utilizes catch bonds to decrease the rolling velocities of RPMI 8226 cells, and rolling regularity can be increased by increasing the shear stress threshold to an optimal level. These results indicate that catch bonding is a key molecular mechanism in the integrin α4β7-mediated flow-enhanced adhesion of RPMI 8226 cells.

The transient tether lifetime measurements from the flow chamber experiment at low shear forces revealed an unstable fluctuation (Figure 3A), which may have occurred due to interference from other sliding cells. Under low shear stress levels, the tethered and sliding cells were indistinguishable. In contrast to the continuously stable rolling cells (Figure 5A), tethered cells were more difficult to distinguish from sliding cells, which resulted in the instability of the obtained data. This effect also resulted in a seemingly triphasic lifetime pattern (Figure 3A). In the case of selectins and integrins, the representative family of adhesion molecules (Table S3), we observed both biphasic and triphasic lifetime curves in different molecules and even within the same molecule type, as in the case of E-selectin [27,29,31], suggesting the possibility of a triphasic lifetime curve in our integrin α4β7–MAdCAM-1 system. To clarify this, we conducted AFM experiments to measure the single-molecule lifetime (Figure 3D), and the results obtained were mutually verified to enhance the reliability of our conclusions regarding the biphasic lifetime and velocity curves.

We examined the integrin α4β7-mediated tether and rolling adhesion behaviors of RPMI 8226 cells in the presence of Ca^2+^ or Mg^2+^ as well, and observed that different ions had different effects on cell-adhesion behavior, which may be attributed to the integrin activation state. The importance of Ca^2+^ for integrin α4β7-mediated initial adhesion and Mg^2+^- and Mn^2+^-mediated firm adhesions has been determined previously [17,18]. However, in this study, we found that the RPMI 8226 cells were mostly firmly adhered in the presence of Mn^2+^, while most other cells exhibited initial phase and rolling adhesion in the presence of Ca^2+^ and Mg^2+^. Moreover, the firm adhesion observed in the presence of Mg^2+^ was significantly higher compared with that observed in the presence of Ca^2+^, which may be due to the activation of integrin α4β7 to an intermediate-affinity state upon binding to Mg^2+^ ions (Figure 2A). In the presence of Mn^2+^, integrin α4β7 was activated to a high-affinity state. The peak in Figure 3A of the adhesion event profile indicates the Mg^2+^-mediated activation of integrin α4β7 at an intermediate-affinity state. Under flow shear force, integrin α4β7 was found to be activated by different ion types into different conformations, which resulted in different affinity states. Therefore, the conformational changes and the signaling pathways involved in the ion-mediated activation of integrin α4β7 need to be studied further.

Regarding the binding of integrin α4β7 to metal ions, it is worth mentioning that α4β7 belongs to the group of integrins lacking αI. The heads of these αI-less integrins are composed of the β propeller domain (α subunit) and βI domain (β subunit), whereas αI-containing integrins have an additional αI domain (α subunit) at the head (Table S2). The βI domain forms a major ligand-binding pocket in αI-less integrins [48,49,50]. In contrast to the αI domain with only one MIDAS, the βI domain contains an interlinked linear array of three metal ion-binding sites, with a MIDAS at the center flanked by two other sites: SyMBS and ADMIDAS [51]. To investigate the effect of divalent metal ions on integrins, the treatment conditions employed herein not only included co-stimulation with multiple metal ions, such as Ca^2+^ and Mg^2+^, but also single-ion stimulation with Ca^2+^, Mg^2+^, or Mn^2+^ alone [14,17,18]. The addition of low concentrations of Ca^2+^ greatly increased the adhesion ability of cells under Mg^2+^ stimulation (Table S1). This effect of enhanced adhesion persisted when the ADMIDAS was mutated, but disappeared when the SyMBS was mutated, indicating that a positive regulation by low concentrations of Ca^2+^ required SyMBS but not ADMIDAS. Both Ca^2+^ and Mg^2+^ tend to bind at the SyMBS and MIDAS, and Ca^2+^ and Mn^2+^ binding to the same ADMIDAS can result in negative or positive effects, and competition with Ca^2+^ can alter the firm adhesion stimulated by Mn^2+^ to rolling adhesion [17].

Considering the role of cytokines in cell adhesion under flow, we selected the chemokine CCL25 as an additional stimulus factor to test alongside the metal ion stimulation. Our objectives were to examine the effect of CCL25 on the adhesion behavior of lymphocytes mediated by the integrin α4β7–MAdCAM-1 interaction and to elucidate the role of CCL25 in the activation of integrin α4β7. In the presence of metal ions, CCL25 enhanced the activity of the integrin (Figure 6), promoted the binding of α4β7 to MAdCAM-1, and resulted in a more robust adhesion. These findings also suggest that chemokine CCL25 and metal ions activate α4β7 and disrupt cell behavior via similar mechanisms. Such similarities may also extend to those of other chemokines and integrins.

In conclusion, the PPFC and ATM experiments were performed to investigate the mechanisms of interaction between integrin α4β7 and MAdCAM-1 in vitro. We found that, similar to selectins, integrin α4β7 displays flow-enhanced rolling adhesion behavior, which is regulated by a catch-bond mechanism. These results improve our mechanistic understanding of chronic IBDs mediated by integrin α4β7 and may inform future efforts in the development of novel anti-IBD drugs targeting integrin α4β7.

Despite the importance of these findings, our study had some limitations. We primarily focused on investigating the force–chemical kinetic mechanisms mediated by force and molecular interactions, which, though interesting, were not fully understood. Therefore, we did not fully consider the issue of biological replication when utilizing multiple cell lines for experimental exploration. In future studies, we plan to incorporate biological replicates to improve the integrity of the study and robustness of its findings. Furthermore, although we used antibodies against binding molecules to improve the orientation of molecular immobilization in the AFM experiments, physisorption was used for the immobilization of most of our molecules (including antibodies), which could lead to ligand orientation problems and affect the efficiency of molecular binding in the experiment, thereby affecting the frequency of adhesion. Based on a study by Gao et al. [52], it may be worthwhile to explore the use of IgG-binding proteins, such as proteins A and G, as linkers to assist in the immobilization of molecules. Furthermore, covalent immobilization mediated by biotin/(strept)avidin may also represent a promising approach to improve the stability of molecular immobilization in future experiments.

## 4. Materials and Methods

### 4.1. Proteins and Cells

The recombinant human MAdCAM-1 Fc chimera and the recombinant human integrin α4β7, a disulfide-linked homodimer, were purchased from R&D Systems (Minneapolis, MN, USA). Human integrin β7 monoclonal antibody (mAb) (MAB4669), human integrin α4 mAb (MAB1354), and recombinant human CCL25/TECK protein (9046-TK) were also purchased from R&D Systems. MAdCAM-1 mAb (F-6, sc-374398) was purchased from Santa Cruz Biotechnology (Santa Cruz, CA, USA). The anti-human IgG antibody (I6260) and anti-6xHis antibody (SAB4301134) were purchased from Sigma Aldrich (St. Louis, MO, USA). The human B-cell line, RPMI 8226, was purchased from the cell bank of the Chinese Academy of Sciences. The RPMI 8226 cells were cultured in RPMI 1640 medium supplemented with 100 U/mL of penicillin, 10 mg/mL of streptomycin, and 10% fetal bovine serum.

### 4.2. PPFC Assay

**Figure 7:**
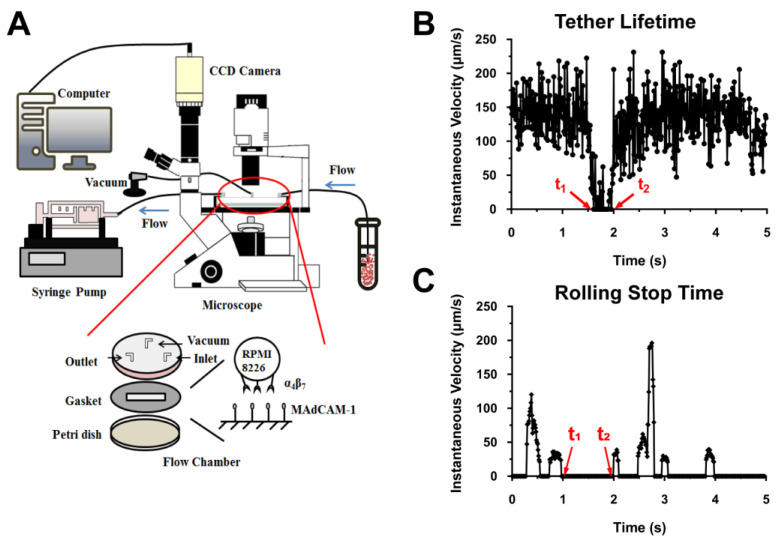
Schematic diagram of the flow chamber device and substrate functionalization experiment. (**A**) The flow chamber device mainly consisted of an organic glass cover, a gasket, and a Petri dish. The upper surface featured three pipe interfaces on the organic glass cover, the vacuum pipe interface in the middle, and separate inlets and outlets on both sides. A grooved ring under the cover was used for fixing the cover onto the Petri dish through vacuuming. The gasket was used to create a space (length × width × height = 2 cm × 0.5 cm × 0.01 in) for the suspension of fluid containing RPMI 8226 cells. At the bottom, a circular polystyrene cell culture dish with a matching organic glass cover was used. MAdCAM-1 was spread and adsorbed onto the surface of the dish to simulate the adherent molecules on the surface of vascular endothelial cells. (**B**) Instantaneous velocity of a transient tethering cell. (**C**) Instantaneous velocity of a rolling cell.

The flow chamber device (Figure 7A) consisted of a Petri dish, a gasket, and a chamber cover with three outfalls (outlet, inlet, and vacuum). The substrate was first functionalized with MAdCAM-1 (alone or together with CCL25) molecules, and a coating region (25 mm^2^) was marked in the center of each 35 mm Petri dish with a clean silicon rubber. Subsequently, a 40 μL solution of MAdCAM-1 (alone or together with CCL25) molecules was added to the marked region and incubated for 12 h at 4 °C. Concentrations of 0.25 and 2 μg/mL of MAdCAM-1 were used for the transient tethering and rolling adhesion experiments, respectively. After incubation overnight, the excess MAdCAM-1 solution was removed and the coated region was washed three times with PBS. To block the non-specific binding sites, Hank’s balanced salt solution (HBSS) containing 2% BSA was added to the coating region and incubated at 24 °C for 1 h.

The RPMI 8226 cells were washed with Ca^2+^- and Mg^2+^-free HBSS containing 10 mM of EDTA. Subsequently, the cells were resuspended in Ca^2+^- and Mg^2+^-free HBSS containing 2% BSA to a final concentration of 5 × 10^5^ cells/ mL. To stimulate the integrin to different activation states, divalent metal ions (1 mM Ca^2+^ and/or 1 mM Mg^2+^ and 0.5 mM Mn^2+^) were added to the cell suspension, which was pumped into the chamber using a syringe pump (Harvard PHD22/2000; Harvard Apparatus, Holliston, MA, USA). During the experiment, various flow shear stress τ_w_ levels (0.05–0.6 dyn/cm^2^) were selected by setting the correspondent flow rate with the syringe pump. The adhesion of the integrin α4β7-expressing RPMI 8226 cells on the substrate coated with MAdCAM-1 (alone or together with CCL25) was observed using an inverted microscope (Axio Observer A1; Zeiss, Jena, Germany) at a 10× magnification. Images were captured using a high-speed CMOS acquisition system (Figure 7A). To block the integrin β7 or α4 subunits, the RPMI 8226 cell suspension was pre-incubated with anti-β7 mAb (MAB4669) or anti-α4 mAB (MAB1354) solutions for 2 h at 37 °C. For MAdCAM-1 blocking, the MAdCAM-1-coated substrate was incubated with anti-MAdCAM-1 mAb (F-6) at room temperature for 1 h. Under each blocking condition, adhesion frequencies in the initial 3 min were measured in the presence of Ca^2+^ at a shear stress of 0.3 dyn/cm^2^. Furthermore, we confirmed the expression of integrin α4β7 on the surface of RPMI 8226 cells (Figure S2) through a flow cytometry experiment involving the incubation with integrin α4β7 mAb (MAB4669).

### 4.3. Transient Tether Lifetime Measurements and Rolling Step Analyses

Under different wall shear stress values, RPMI 8226 cells were perfused into the flow chamber, where they tethered transiently to the low-density MAdCAM-1-coated substrate via single integrin α4β7–MAdCAM-1 bonds. Images of transient tethers were captured using a high-speed CMOS at 100 fps (frames per second). These frames were replayed in slow motion, and the duration of each transient tether was measured via a frame-by-frame analysis. The average lifetime under each wall shear stress was measured as approximately 60 tether events. A tether event was defined as a stop duration (t_1_–t_2_), where the instantaneous speed of a sliding cell dropped to zero, and then returned to the original sliding speed (Figure 7B). The tether lifetime was thus analyzed using the natural log of (number of lifetimes ≥ t) versus time (t) plot. The negative slope of the linear fit to this distribution was regarded as the dissociation rate, *k_off_*. The dissociation rates were then plotted against wall shear stress [27,29].

The analysis of the cell-rolling behavior was conducted using a stop-and-go model [31,43], which assumed that the rolling of cells only depended on the conversion between two adhesive molecule bonds. When a front bond forms, the cell decelerates and enters the stop phase. Once a trail bond dissociates, the cell accelerates and enters the go phase, while the previous front bond becomes the trail bond. Therefore, the cell-rolling process comprises many stop and go phases. In the instantaneous velocity diagram, after calculating the sliding average for every 10 frames (Figure 1C), if the cell instantaneous rolling velocity was lower than the average noise level of the image system (25 μm/s), the phase was defined as the stop phase and the velocity was set to 0 μm/s; otherwise, the phase was defined as the go phase (Figure 7C). In our study, the rolling of RPMI 8226 cells was tracked frame-by-frame (100 fps) using Image Pro Plus software (version 6.0). Cells with a stable rolling adhesion were used for the collection of the cell-rolling data. Subsequently, the data for approximately 100 stop-and-go events of 15–30 RPMI 8226 cells rolling continuously on a high-density MAdCAM-1 substrate for 5 s at each shear stress were collected. Thereafter, a Microsoft Excel macro (version 2306) was used to statistically analyze these events [43] and describe the rolling behavior of RPMI 8226 cells based on the quantitative parameters, including the mean rolling velocity, mean stop time (the average of the stop time when the targeted cell entered a stop phase), and stop frequency (the average of the number of stops per unit time for a targeted cell in the stop-and-go model). The rolling speed of each cell was calculated as the average rolling speed of 500 frames, with the proper slip averaging of the instantaneous velocity of each frame calculated using Image Pro Plus software. The calculations of the stop time and stop frequency for each rolling cell were also based on the instantaneous velocity of each frame calculated using Image Pro Plus software and the stop-and-go model [43].

### 4.4. AFM System Setup and Functionalization

**Figure 8:**
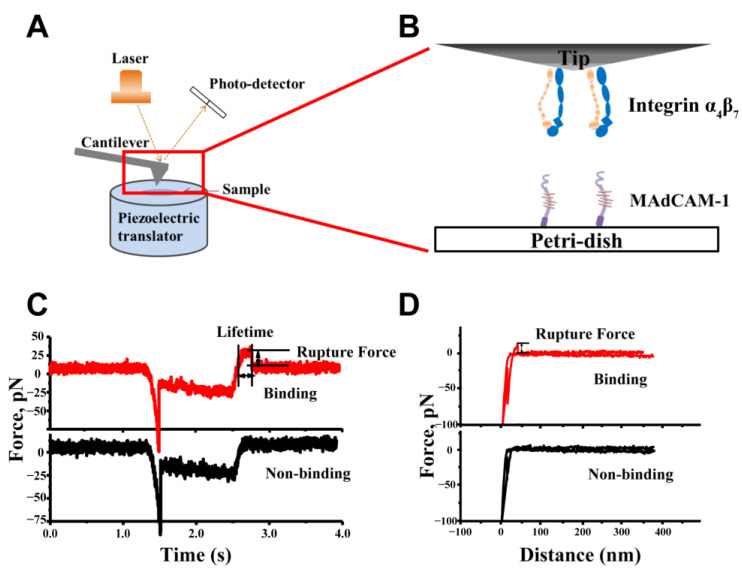
Schematic diagram of the atomic force microscope (AFM) experiment. (**A**) Diagram of the experimental AFM device. (**B**) AFM functionalization. (**C**) Force–time diagram for typical adhesion events. (**D**) Force–distance diagram for typical adhesion events.

A CSPM5500 AFM system (BenYuan, Guangzhou, China) was used in this study (Figure 8). The AFM system was functionalized by coating integrin α4β7 and MAdCAM-1 onto the surface of the dish and the tip of the microscope (Figure 8A,B). Adhering molecules were either captured directly by the surface or anti-6xHis antibodies present on the surface. Each microscope tip (MLCT, Bruker, Billerica, MA, USA) was incubated in 10 μL of MAdCAM-1 (10 μg/mL), anti-IgG (20 μg/mL), or PBS (Gibco, Thermo Fisher Scientific, Waltham, MA, USA) containing 2% BSA at 4 °C overnight, followed by incubation in PBS containing 2% BSA to block non-specific binding for 30 min. In some inhibition experiments with specific antibodies, the tip containing MAdCAM-1 was further incubated with the blocking antibody F-6 (20 μg/mL), while the tip containing anti-IgG was further incubated with MAdCAM-1 (10 μg/mL) on its surface and left at room temperature for 1 h. Before the incubation of the Petri dish with integrin α4β7, the Petri dish was thoroughly washed with anhydrous ethanol and then dried with nitrogen. After drying the Petri dish, 10 μL of integrin α4β7 (10 μg/mL) or anti-6xHis (20 μg/mL) was incubated in the Petri dish overnight at 4 °C, followed by washing with PBS twice and blocking with 2% BSA in PBS on the 2nd day. For some blocking experiments with antibodies, the anti-β7 antibody was further incubated in a culture dish with a substrate of α4β7 (10 μg/mL), and anti-6xHis was further incubated on the substrate of α4β7 (10 μg/mL). To create environments with various ionic conditions, the dish was filled with 10 mM of EDTA, 5 mM of Ca^2+^, 5 mM of Mg^2+^, or 5 mM of Mn^2+^ in PBS containing 2% BSA.

### 4.5. AFM Experiments of the Adhesion Frequency and Lifetime Measurement

The AFM analysis was performed to measure the interaction between integrin α4β7 and MAdCAM-1. During the experiment, an AFM tip was driven to contact the surface of the sample using a piezoelectric translator, where the pressure could be detected using a photo-detector. After detecting the compression force with the photo-detector (<30 pN), the cantilever was retracted by 20 nm and held for 0.5 s to form a bond [53]. Subsequently, the cantilever was retracted again until the bond dissociated, and the strength of the tension was measured using the photo-detector. This process was defined as a test of adhesion. Here, the calculation of the strength depended on the spring constants of the needle tip, which were evaluated using the thermal wave method [43,54]. According to the manufacturer’s instructions, the spring constant at the needle tip was within a reasonable range of 15–30 pN/nm. During each measurement cycle, the functionalized (MAdCAM-1) or non-functionalized (2% BSA) tip was brought into contact with a point coated with protein or 2% BSA for 0.5 s and retracted to its original position. At this point, a significant increase in the decrease in the voltage signal was considered an adhesion event. Subsequently, the adhesion frequency and lifetime were analyzed (Figure 8C,D). More than three different contact points were detected in each sample. An adhesion frequency of non-specific interaction less than 7% was considered effective.

### 4.6. Statistical Analysis

The *p*-values from unpaired two-tailed Student’s *t*-tests and unpaired two-way ANOVA for multiple comparisons were used to determine the statistical difference significance of the data (* for *p* < 0.05, ** for *p* < 0.01, *** for *p* < 0.001).

## Data Availability

Data are contained within the article and Supplementary Materials.

## Supplementary Materials

The following supporting information can be downloaded at: https://www.mdpi.com/article/10.3390/ijms242216062/s1. References [55,56,57,58,59] are cited in Supplementary Materials.
