# Supplementary material for: The Force-Dependent Mechanism of an Integrin α4β7–MAdCAM-1 Interaction
Source: Int J Mol Sci. 2023 Nov 7;24(22):16062. doi: 10.3390/ijms242216062 (PMC10670920; doi:10.3390/ijms242216062)
Supplement: Supplementary file 1 [file ijms-24-16062-s001.zip › ijms-2636412-supplementary.pdf]

International Journal of
Molecular Sciences

MDPI

Supplementary Materials

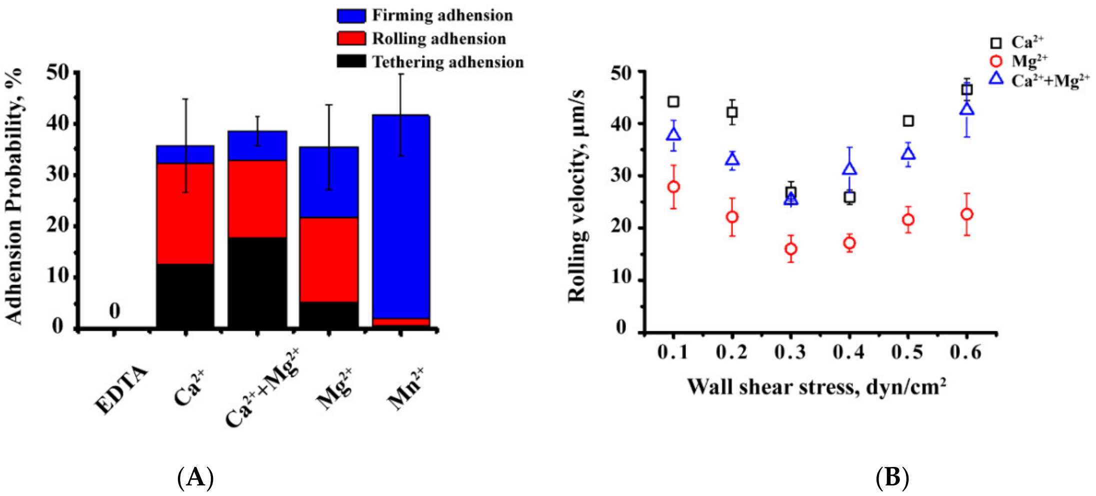

Figure S1. Adhesion probability and rolling velocity of cells in different metal ion solutions. (A) Probability of cell adhesion. (B) Rolling velocity of different adhesion behaviors.

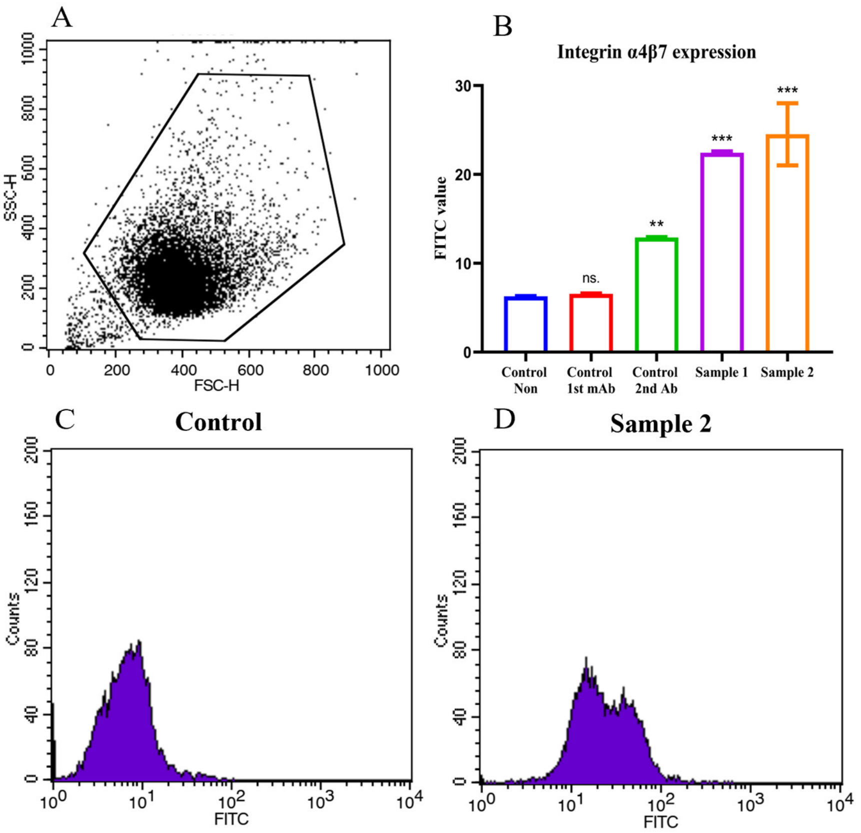

Figure S2. Measurement of integrin $\alpha 4\beta 7$ expression. (A) Dot plots; (B) FITC value of integrin $\alpha 4\beta 7$ expression. The Control Non, Control 1st mAb (MAB4669), Control 2nd Ab, and Sample 1 and 2 groups were compared with the RPMI 8226 cells incubated with nothing, only 1st mAb, only 2nd Ab, and both 1st mAb and 2nd Ab. (C) Histogram for Control Non group. (D) Histogram for Sample 2 group. ns for no significant difference, ** for $p < 0.01$, *** for $p < 0.001$.

Table S1. Metal ion-binding sites in integrin α4β7 [17].

Metal ion-binding site	Divalent metal cations	Detail
MIDAS	Ca^{2+}	Rolling adhesion
	Mg^{2+}	Trend of firm adhesion
		Mg^{2+} binds with MIDAS upon co-stimulation with low concentrations of Ca^{2+} and Mg^{2+}
	Mn^{2+}	Firm adhesion
ADMIDAS	Ca^{2+}	High concentration of Ca^{2+} binding to ADMIDAS negatively regulates integrin α4β7
	Mn^{2+}	Mn^{2+} activates integrins by competing with Ca^{2+} at ADMIDAS
SyMBS (LIMBS)	Ca^{2+}	Low concentration of Ca^{2+} positively regulates integrin α4β7 to stabilize cell adhesion
		Low concentrations of Ca^{2+} and Mg^{2+} bind to SyMBS and MIDAS, respectively

Table S2. Different characteristics of metal ion-binding sites between integrins with and without α.

Binding sites	αI integrins	Non-αI integrins
MIDAS (αI domain)	Located on the top face of the αI domain [55,56]<br>Affinity for metal ions: $Mn^{2+} > Mg^{2+} > Ca^{2+}$ [55,57]	NA
MIDAS (βI domain)	Binding to the activated αI domain of MIDAS as an intrinsic ligand [55], propensity to bind Mg^{2+}	The activation of integrins by the three divalent metal ions, Ca^{2+}, Mg^{2+}, and Mn^{2+}, requires binding to MIDAS [17]
ADMIDAS (βI domain)	The negative regulatory site under a high concentration of Ca^{2+}, Mn^{2+} competes for this site with Ca^{2+} to activate integrin [17,58,59].	Identical feature
SyMBS (βI domain)	Positive regulatory site prefers to bind Ca^{2+} [17]	Identical feature

Table S3. Lifetime curves of selectins and integrins.

	Receptor	Ligand	Lifetime curve
Selectin family	P-selectin	PSGL-1	Biphasic shape
	L-selectin	PSGL-1	Biphasic shape [28,30]
	E-selectin	Ligands on HL-60	Biphasic shape [27]; triphasic [29,31]
Integrin family	LFA-1	ICAM-1	Biphasic shape [46]
	α5β1	Fibronectin	Biphasic and triphasic [43]
